# Highly Heterogeneous Bacterial Communities Associated with the South China Sea Reef Corals *Porites lutea*, *Galaxea fascicularis* and *Acropora millepora*

**DOI:** 10.1371/journal.pone.0071301

**Published:** 2013-08-07

**Authors:** Jie Li, Qi Chen, Si Zhang, Hui Huang, Jian Yang, Xin-Peng Tian, Li-Juan Long

**Affiliations:** 1 CAS Key Laboratory of Tropical Marine Bio-resources and Ecology, RNAM Center for Marine Microbiology, South China Sea Institute of Oceanology, Chinese Academy of Sciences, Guangzhou, Guangdong, P. R. China; 2 Graduate University of Chinese Academy of Sciences, Beijing, P. R. China; Rockefeller University, United States of America

## Abstract

Coral harbor diverse and specific bacteria play significant roles in coral holobiont function. Bacteria associated with three of the common and phylogenetically divergent reef-building corals in the South China Sea, *Porites lutea*, *Galaxea fascicularis* and *Acropora millepora*, were investigated using 454 barcoded-pyrosequencing. Three colonies of each species were sampled, and 16S rRNA gene libraries were constructed individually. Analysis of pyrosequencing libraries showed that bacterial communities associated with the three coral species were more diverse than previous estimates based on corals from the Caribbean Sea, Indo-Pacific reefs and the Red Sea. Three candidate phyla, including BRC1, OD1 and SR1, were found for the first time in corals. Bacterial communities were separated into three groups: *P*. *lutea* and *G*. *fascicular*, *A. millepora* and seawater. *P*. *lutea* and *G*. *fascicular* displayed more similar bacterial communities, and bacterial communities associated with *A. millepora* differed from the other two coral species. The three coral species shared only 22 OTUs, which were distributed in *Alphaproteobacteria*, *Deltaproteobacteria*, *Gammaproteobacteria*, *Chloroflexi*, *Actinobacteria*, *Acidobacteria* and an unclassified bacterial group. The composition of bacterial communities within each colony of each coral species also showed variation. The relatively small common and large specific bacterial communities in these corals implies that bacterial associations may be structured by multiple factors at different scales and that corals may associate with microbes in terms of similar function, rather than identical species.

## Introduction

The abundance of bacteria has been shown to be an important part of the coral holobiont [1]. Coral-associated bacteria are ubiquitous in the coral holobiont temporally and spatially. Planulae older than 79 h harbor internalized bacteria cells [2]. Subsequently, abundant and various bacterial communities were associated with adult corals, for example, *Stylophora pistillata* and *Pocillopora damicornis* [3], [4]. Evidence has also accumulated suggesting that coral-associated bacterial communities respond to dynamic environmental conditions at different scales [5]–[7]. In divergent compartments of corals, such as mucus, tissues and the calcium carbonate skeleton, dissimilar bacteria communities have been detected [8], [9]. Although coral-associated bacterial communities are diverse, they are distinct from ambient seawater bacterial communities [1], [10], [11]. Diverse and dynamic coral-associated bacteria assemblages potentially have functions related to nitrogen, carbon and sulfur metabolism, coral disease resistance and abiotic stress tolerance [12].

Our understanding of the specificity of coral-associated microorganisms is changing because the information on coral-derived microbial sequences is increasing at a staggering rate. The bacterial communities associated with the corals *Montastraea franksi*, *Diploria strigosa* and *Porites astreoides* from Panama and Bermuda [1] support the argument that coral-associated bacterial assemblages are most likely species-specific. In contrast, Littman et al. [13] reported that the bacterial communities in three species of *Acroporid* corals on the Great Barrier Reef were more crucially shaped by location than by the host coral species. Meanwhile, the argument that coral bacterial communities may be both site and species specific has been recently reported [14]. Although all of these studies support the conclusion that corals possess specific microbiota, the inconsistency of the findings on specificity across studies should not be overlooked. These results were mainly obtained using conventional cloning and sequencing or DGGE methods. Therefore, the major limitation of these studies is that the characterization of the microbial communities is not comprehensive. More recently, pyrosequencing has been employed to investigate the bacterial community associated with corals [11], [15]–[19]. These studies have further supported the conclusion that the bacterial communities appear to be regulated by the host coral species. In addition to the coral species, the significant influence derived from environmental factors has also been emphasized. Therefore, the specificity of coral bacterial communities is more complex than initially thought and still obscure. To better understand the nature of the specificity of coral-associated microorganisms, more comprehensive surveys about more corals from different environments at different scales are required.

Coral reefs are widely distributed across the South China Sea, with a total reef area of approximately 7974 km^2^, matching the Great Barrier Reef in size, latitudinal range and biodiversity [20]. However, the microbial consortium has been rarely documented in South China Sea corals. The Luhuitou fringing reef located in Sanya, southern Hainan Island, is approximately 3500 m long and 250–500 m wide and consists of approximately 70% of the coral species so far reported for Hainan Island and its surrounding islands [20]. Luhuitou is a popular tourist location; therefore, investigating of the bacteria associated with local coral colonies is crucial for us to estimate anthropogenic impacts on the coral reef. The aim of this study is to comprehensively investigate the diversity and structure of bacterial communities associated with the three dominant coral species *Porites lutea*, *Galaxea fascicularis* and *Acropora millepora* from the South China Sea. Furthermore, we compared the bacterial communities among coral species and individual colonies to define the common and specific bacteria communities in these corals. Such information will provide a further understanding of the specificity of coral-associated bacteria.

## Materials and Methods

### Ethics Statement

Permits for coral sampling were provided by the Administration of Sanya Coral Reef National Nature Reserve, the Department of Ocean and Fisheries of Hainan Province.

### Sample Collection

Coral and seawater samples were collected in July 2011 from the Luhuitou fringing reef (18°13′N, 109°28′E), Sanya, Hainan province, China. Three coral species, including *P. lutea*, *G. fascicularis* and *A. millepora*, were sampled at a depth of 3–5 m using a punch and hammer. The temperature of ambient sea water was approximately 27–28°C, the average pH was 8.78±0.01, and the salinity was 34. Triplicate samples of each species were collected. The interval distance of sampling was 0.5 m. All nine samples were washed with autoclaved sea water and then placed in sterile plastic bags. Ambient sea water was collected into sterile plastic bottles and then filtered through a 0.22-µm polycarbonate filter membrane (Millipore). All samples were frozen at −80°C until DNA extraction.

### DNA Extraction, PCR Amplification and Pyrosequencing

The coral samples were homogenized in liquid nitrogen with a mortar and pestle. The 0.22-µm polycarbonate filter membranes with adsorbed microbial cells were cut into pieces before DNA extraction. Total DNA was extracted using the PowerSoil DNA Isolation Kit (MoBio, Solana Beach, CA, USA) according to the manufacturer’s instructions. Bacterial V1–V3 hypervariable regions of the 16S ribosomal RNA gene were amplified using the bacterial forward primer 27F [21], which includes the primer A adaptor and a unique 10 bp barcode on the 5′ end (5′-CCATCTCATCCCTGCGTGTCTCCGACTCAGNNNNNNNNNNAGAGTTTGATCCTGGCTCA-3′), and the reverse primer 534R with the primer B adaptor on the 5′ end (5′-CCTATCCCCTGTGTGCCTTGGCAGTCTCAGCAATTACCGCGGCTGCTGG-3′) [22]. PCR amplifications were performed in a Mastercycler pro (Eppendorf, Hamburg, Germany) in a final volume of 50 µl, containing 4 µl of 2.5 mM deoxynucleotide triphosphate mixture (TaKaRa), 2 µl of 10 µM each primer, 5 µl (10–20 ng) template DNA and 2.5 units *Ex Taq* DNA polymerase (TaKaRa, with its recommended reaction buffer). The PCR conditions were as follows: 94°C for 5 min; 30 cycles of 94°C for 30 s, 60°C −0.5°C/cycle for 30 s, 72°C for 30 s; followed by 72°C for 10 min. Each genomic DNA sample was amplified in triplicate PCR reactions, and amplicons were pooled and purified using the E.Z.N.A.® Gel Extraction Kit (Omega Bio-Tek). The quality of the purified PCR products was assessed using a Nanodrop spectrophotometer (Thermo Scientific, Vantaa, Finland). Pooled 200 ng of the purified tagged amplicons from each sample were pyrosequenced on the Roche 454 Genome Sequencer FLX System.

### Pyrosequencing Analysis

After excluding the reads with low quality scores (<20) and containing homopolymer inserts, high quality reads were reserved for downstream analysis [23]. The pyrosequencing data were deposited in the NCBI Sequence Read Archive (SRA) database under the accession number SRP020939. Chimeras were detected by running chimera.uchime packaged in Mothur [24], and potential chimeras were removed. All quantified sequences were identified using the RDP classifier with a bootstrap confidence level of 50% [25]. Sequences were clustered into operational taxonomic units (OTUs) with a 97% threshold using uclust [26]. Species richness and diversity estimates were performed using Mothur [24]. To standardize all datasets, the smallest number (678) of sequences was randomly selected from each sample 1000 times. The relationships among bacterial assemblages of coral and seawater samples were analyzed by non-Metric Multidimensional scaling (nMDS) ordination [27]. The Bray-Curtis distance matrix was estimated from the OTU matrix, and then, the nMDS profile was generated by the PRIMER 5 software (PRIMER-E, Lutton, Ivybridge, UK). Differences in bacterial communities between categories were tested with an analysis of similarities (ANOSIM), with 10,000 replicates [27]. SIMilarity PERcentage (SIMPER) analysis was carried out to determine which taxa generated the most differences between categories [27].

## Results

### Diversity of Coral-associated Bacteria

A total of 29877 reads were recovered through quality filtering and clustered into 8316 different 97% OTUs. The number of reads ranged from 678 to 6450 in each sample. The largest number of 1031–2463 OTUs was in *P. lutea* compared to the 646–1459 and 361–724 OTUs that were associated with *G. fascicularis* and *A. millepora*, respectively (Table 1). The bacterial communities associated with the three coral species were highly diverse. The Shannon index ranged from 5.79 to 7.04 in *P. lutea*, from 4.16 to 6.72 in *G. fascicularis* and from 5.26 to 5.83 in *A. millepora*. The value of the Shannon index in sea water was 4.95. As the *P*-value was 0.14 to 0.67, there was no significant difference in the Shannon index among corals and seawater samples.

**Table 1:** Numbers of sequences and OTUs (97%) and diversity estimates of coral-associated bacteria.

Index	*Porites lutea*			*Galaxea fascicularis*			*Acropora millepora*			Seawater
	colony 1	colony 2	colony 3	colony 1	colony 2	colony 3	colony 1	colony 2	colony 3	
No. of Seq	4,780	2,857	6,450	2,839	2,766	3,249	2,085	678	1,078	3,093
OTUs	1374	1031	2463	1459	1438	646	724	361	484	788
Chao 1	2993.29	2878.81	5671.05	4350.69	4663.02	1597.86	1261.98	775.75	1392.47	2073.51
ACE	4515.78	5673.82	8970.90	7518.84	8547.68	2666.94	1642.64	855.53	2368.62	3584.98
Shannon	6.03	5.79	7.04	6.63	6.72	4.16	5.83	5.46	5.26	4.95

### Bacterial Community Composition

**Figure 1:**
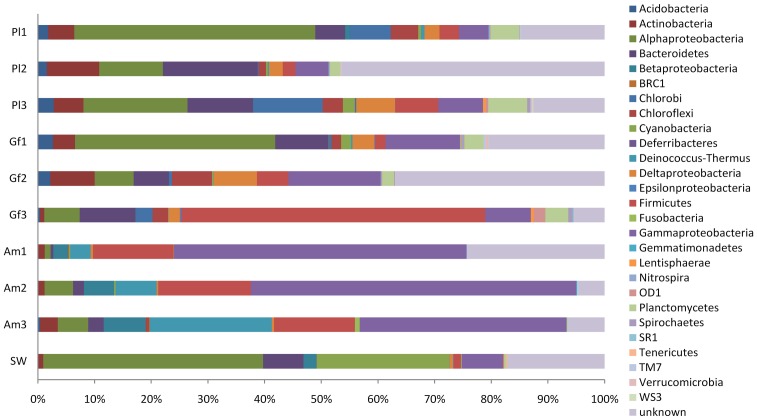
Bacterial composition profiles. Pl, *Porites lutea*; Gf, *Galaxea fascicularis*; Am, *Acropora millepora*; SW, seawater.

At a confidence threshold of 50%, 24135 out of the 29877 qualified reads could be assigned to 18 formally described bacterial phyla and 5 candidate phyla (Fig. 1).?The proportions of these phyla varied among different coral species and seawater libraries. *Alphaproteobacteria* were predominant in the *P. lutea* (11.2–42.5%) and *G. fascicularis* (6.3–35.3%) libraries compared to *A. millepora* (1.1–5.4%). Within *Alphaproteobacteria*, *Silicibacter* were predominant in the *P. lutea* and *G. fascicularis* libraries, while no sequence belonging to the genus *Silicibacter* was detected in the *A. millepora* libraries. Additionally, *Sphingomonas* was a major group in the *P. lutea*-associated bacterial community, which was not present in the *G. fascicularis* libraries. Classified reads affiliated with *Alphaproteobacteria*, especially *Rhodobacterales*, were also the most abundant group in the seawater library (38.8%). More sequences affiliated with *Bacteroidetes* were detected in the *P. lutea* (5.3–16.7%), *G. fascicularis* (6.2–9.9%) and seawater libraries (7.1%) in contrast to the *A. millepora* (0.5–2.7%) libraries. Both *Flavobacteria* and *Sphingobacteria* were the major *Bacteroidetes* groups in coral samples, while *Flavobacteria* were more abundant than *Sphingobacteria* in the seawater library. *Prosthecochloris* was the most abundant *Chlorobi* group in *P. lutea* colonies 1 and 3, but *Prosthecochloris* was much rarer in the *G. fascicularis* libraries and was not even present in the *A. millepora* and seawater libraries. *Betaproteobacteria* were more predominant in the *A. Millepora* (2.5–7.4%) and seawater (2.3%) libraries compared to the *G. fascicularis* and *P. lutea* (<0.8%) libraries. *Burkholderiales* were the major *Betaproteobacteria* group in the *A. millepora* and seawater libraries. A total of 1.8–6.9% and 2.1–4.0% of sequences were related to *Planctomycetes* in the *P. lutea* and *G. fascicularis* libraries, respectively, but were not detected in the *A. millepora* libraries.

*Firmicutes* were more predominant in *A. millepora* (14.2–16.4%) compared to *P. lutea* (2.3–7.6%) and *G. fascicularis* colony 1 (1.8%) and colony 2 (5.6%), and were much rarer (1.3%) in the seawater library. In *G. fascicularis* colony 3, the spike of *Firmicutes* (53.7%) resulted from an increasing presence of *Lachnospiraceae*. *Gammaproteobacteria* (36.6–57.4%), primarily *Enterobacteriales*, dominated the *A. millepora* libraries compared to the *G. fascicularis* (8.0–16.3%), *P. lutea* (5.2–7.9%) and seawater (7.3%) libraries. In addition to *Gammaproteobacteria* and *Firmicutes* groups, *Deinococci* was another major group in the *A. millepora* bacterial community that was rarely detected in the *G. fascicularis*, *P. lutea* and seawater libraries.

Other differences in coral-associated bacteria included *Acidobacteria*, which account for 1.6–2.8% of the bacteria in the *P. lutea* libraries and in *G. fascicularis* colonies 1 and 2 (0.34% in *G. fascicularis* colonies 3) but were absent from the *A. millepora* and seawater libraries with the exception of 0.37% in *A. millepora* colony 3. Similarly, relatively abundant sequences related to *Deltaproteobacteria* were found in the *P. lutea* (2.4–6.8%) and *G. fascicularis* (2.0–7.6%) libraries, which were rarely detected in *A. millepora*. *Actinobacteria* were abundant in coral libraries, especially in *P. lutea* and *G. fascicularis*, but were rare in the seawater library. Five candidate phyla, including BRC1, OD1, SR1, TM7 and WS3, were detected in the coral data set. Among them, OD1 and WS3 were present in both *P. lutea* and *G. fascicularis*, and TM7 were detected in all three types of corals, while BRC1 and SR1 were only observed in *G. fascicularis*. In the seawater library, 23.4% of sequences were related to *Cyanobacteria* compared to <2.1% in the coral libraries.

### Comparison of Coral-associated Bacterial Communities

**Figure 2:**
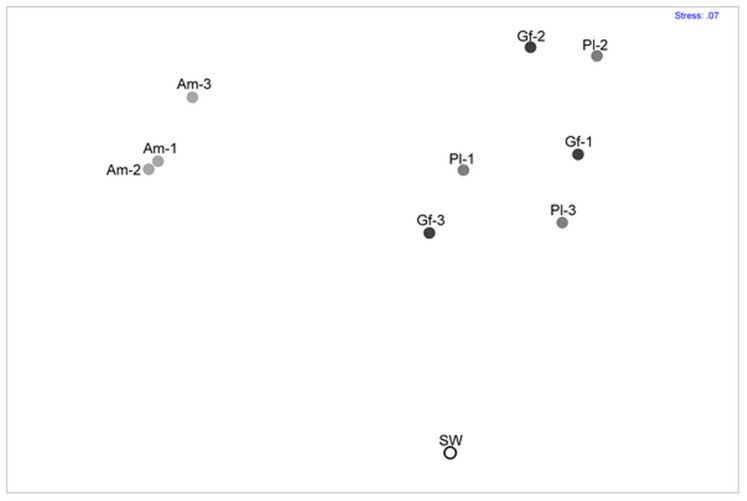
Non-metric multidimensional scaling plot showing the distance of each sample. Pl, *Porites lutea*; Gf, *Galaxea fascicularis*; Am, *Acropora millepora*; SW, seawater sample.

The nMDS matrix was generated from the OTU percentages in each sample and was computed to compare the similarity of the bacterial communities among different coral species (Fig. 2). Bacterial communities from *P*. *lutea* and *G*. *fascicular*, *A. millepora* and seawater showed significant differences between each other in global testing (*R* = 0.99, *P* = 0.001). Bacterial communities associated with *P*. *lutea* and *G*. *fascicular* did not show significant difference (*P*>0.05), and they were separated from the *A. millepora*-associated bacterial communities (*R* = 0.99, *P* = 0.012). Such differences in bacterial community composition between *P*. *lutea* and *G*. *fascicular* and *A. millepora* are caused by those taxa that are restricted to either *P*. *lutea* and *G*. *fascicular* or *A. millepora* (Table S1).

### The Distribution of Ubiquitous and Unique Bacterial Groups

The distribution of OTUs within the coral samples was investigated by combining all tag sequences and determining their presence in different coral species. All of the three coral species shared only 22 97% OTUs, 14 of which were distributed in *Alphaproteobacteria*, *Deltaproteobacteria*, *Gammaproteobacteria*, *Chloroflexi*, *Actinobacteria* and *Acidobacteria*, whereas the other 8 could not be identified as any described bacterial group using the RDP classifier at a confidence level of 50% (Table 2). These OTUs presented at all three coral species were defined as a common community. In contrast to the common bacterial community, the species-specific community is very large. There were 3448, 2350 and 886 unique OTUs observed in *P. lutea*, *G. fascicular* and *A. millepora*, respectively.

**Table 2:** Common bacterial community in corals.

Tag ID	Taxonomic affiliation	*Pl*	*Gf*	*Am*	Best BLAST-hit description					
					score	coverage %	E-value	identity %	accession #	source
HEW737K01CVO0E_MID45	*Bacteria*	21#	26	1	867	99	0	98	FJ481263	uncultured *Chloroflexus* sp. from sponge (*Xestospongia testudinaria*) tissue, Indonesia
HEVCZ0I01CQ4LC_MID44	*Bacteria*	16	3	1	809	99	0	96	FM214050	uncultured bacterium from surface marine sediment, Antarctica:Crozet Island Archipelago
HEVCZ0I01AG4I7_MID48	*Bacteria*	14	23	1	931	100	0	98	GU982049	uncultured bacterium from sponge *Aplysina fulva*, Bahamas: Sweetings Cay, Mangrove
HEVCZ0I01ANRV0_MID45	*Bacteria*	10	9	1	835	99	0	97	JX206718	uncultured bacterium from marine sponge *Ircinia variabilis*, Spain: Catalonia
HEVCZ0I01ER8YY_MID45	*Bacteria*	10	26	1	861	99	0	97	FJ543133	uncultured bacterium from sponge *Acanthostrongylophora* sp., Indonesia: Manado
HEVCZ0I01ARZKL_MID48	*Bacteria*	6	22	1	869	99	0	97	AY897114	uncultured bacterium from sponge *Discodermia dissoluta*
HEVCZ0I01C2XUU_MID47	*Bacteria*	2	6	1	826	96	0	98	GU118631	uncultured bacterium from coral *Montastraea faveolata*,“Crawl Cay” reef
HEW737K01EP96Q_MID47	*Bacteria*	1	1	1	821	99	0	96	DQ395368	uncultured organism from deep-sea octacoral
HEW737K01DSVHN_MID47	*Acidobacteria; Acidobacteria_Gp9; Gp9*	1	1	1	904	99	0	97	JN886924	uncultured *Acidobacterium* sp. from carbonate sediments at the South West Indian Ridge
HEW737K01BY1NL_MID52	*Acidobacteria; Acidobacteria_Gp10; Gp10*	6	2	1	905	100	0	97	FR851552	uncultured bacterium from permeable coral reef sands, the Red Sea
HEVCZ0I01EBSUJ_MID48	*Acidobacteria; Acidobacteria_Gp21; Gp21*	2	3	1	872	99	0	98	JX504510	uncultured bacterium from oolitic sand, the Bahamas: Highborne Cay
HEVCZ0I01DF7YC_MID47	*Actinobacteria; Actinobacteria; Acidimicrobidae; Acidimicrobiales; Acidimicrobineae; Acidimicrobineae_incertae_sedis; Aciditerrimonas*	1	1	1	750	99	0	94	EF018121	uncultured bacterium from trembling aspen rhizosphere under ambient CO_2_ conditions
HEVCZ0I01D460N_MID48	*Chloroflexi; Caldilineae*; *Caldilineales*; *Caldilineaceae*; *Caldilinea*	15	34	1	898	99	0	98	FJ543133	uncultured bacterium from sponge *Acanthostrongylophora* sp., Indonesia: Manado
HEW737K01BEAAP_MID52	*Chloroflexi; Caldilineae; Caldilineales; Caldilineaceae; Caldilinea*	2	31	7	835	99	0	96	FJ543133	uncultured bacterium from sponge *Acanthostrongylophora* sp., Indonesia: Manado
HEW737K01BL9MG_MID49*	*Proteobacteria; Alphaproteobacteria; Rhodobacterales; Rhodobacteraceae; Litoreibacter*	3	17	1	830	99	0	99	EF123356	uncultured alpha proteobacterium from black band diseased *Siderastrea siderea* coral tissues, the Bahamas: Lee Stocking Island, Rainbow Garden Reef
HEVCZ0I01CPO8B_MID44*	*Proteobacteria; Alphaproteobacteria; Rhizobiales; Bradyrhizobiaceae; osea*	3	2	1	843	99	0	99	JX286022	uncultured bacterium from Cincinnati drinking water system
HEW737K01B9GIH_MID45	*Proteobacteria; Deltaproteobacteria*	18	24	1	857	99	0	97	DQ889875	uncultured delta proteobacterium from gorgonian octocoral *Erythropodium caribaeorum*
HEVCZ0I01EZU2I_MID48	*Proteobacteria; Deltaproteobacteria*	11	33	2	891	99	0	98	DQ889875	uncultured delta proteobacterium from gorgonian octocoral *Erythropodium caribaeorum*
HEVCZ0I01AN8PE_MID49	*Proteobacteria; Gammaproteobacteria; Enterobacteriales; Enterobacteriaceae; Escherichia/Shigella*	1	1	8	876	98	0	98	GU535930	uncultured bacterium from Guri wastewater [attached biomass (2)]
HEW737K01CAH9Q_MID49	*Proteobacteria; Gammaproteobacteria; Enterobacteriales; Enterobacteriaceae; Escherichia/Shigella*	1	1	5	896	99	0	98	JN866566	uncultured bacterium from *Haemaphysalis longicornis*
HEW737K01DLRLP_MID50	*Proteobacteria; Gammaproteobacteria; Enterobacteriales; Enterobacteriaceae; Escherichia/Shigella*	1	2	8	869	100	0	97	EU472715	uncultured bacterium from red panda (*Ailurus fulgens*) feces
HEW737K01C6ZJ7_MID50	*Proteobacteria; Gammaproteobacteria; Enterobacteriales; Enterobacteriaceae; Escherichia/Shigella*	1	1	4	828	92	0	97	KC011135	uncultured *Enterobacteriaceae* bacterium from soil

* represents the OTUs present both in corals and seawater libraries.

# numbers show the reads detected in different coral species. *Pl*, *Porites lutea*; *Gf*, *Galaxea fascicularis*; *Am*, *Acropora millepora*.

In the nMDS profile, compositions of bacterial communities within the three colonies of *A. millepora* grouped at the similarity value 33.9%. Analysis of the OTU composition indicated that 11.2% of all OTUs were shared by the three *A. millepora* colonies and were affiliated with all major groups detected in this study, including *Actinobacteria*, *Betaproteobacteria*, *Deinococcus-Thermus*, *Firmicutes*, *Gammaproteobacteria* and unclassified bacterial groups (Table S2 in File S2). The *P. lutea* and *G. fascicularis* bacteria communities grouped at an approximately 9.0% similarity level. Approximately 1.8% of all OTUs were observed in all three colonies of *P. lutea*, and 1.1% of all OTUs were shared by *G. fascicularis* colonies. Similar to OTUs shared by the *A. millepora* colonies, OTUs shared by the *P. lutea* or *G. fascicularis* colonies were distributed in their own dominant groups, i.e., *Acidobacteria*, *Actinobacteria*, *Alphaproteobacteria*, *Bacteroidetes*, *Chlorobi*, *Chloroflexi*, *Firmicutes*, *Deltaproteobacteria*, *Gammaproteobacteria*, *Planctomycetes* and unclassified bacterial groups (Table S3 & S4 in File S2).

## Discussion

### Coral Bacterial Community Analysis

Presently, the high-throughput pyrosequencing technique combined with barcoded PCR primers has been used for the survey of coral-associated bacterial communities by several researchers [10], [11], [15]–[19]. The number of OTUs detected in a single species of coral in the present study is similar to results obtained from *Isopora palifera* collected from Tan-Tzei Bay [11]. In comparison with the results shown by previous studies, this study revealed a higher bacterial diversity in corals from Sanya Bay than those from the Caribbean Sea [10], [17], [18], Indo-Pacific reefs [18] and the Red Sea [19]. This difference may be due to technical factors, including PCR primer selection and sequencing depth, and may still reflect the essential distinction among different coral species in different environments. Sunagawa et al [15] found that mounding corals (*Montastraea faveolata*, *M. franksi*, *D. strigosa* and *P. astreoides*) had higher estimated diversities than branch-forming acroporid corals and, therefore, speculated that coral morphology plays a role in determining the diversity of coral bacteria. In this study, although the estimated diversities of coral-associated bacteria were not significantly different, they were grouped into *A. millepora* or *P. lutea* and *G. fascicularis* categories, which supports the previous hypothesis to a certain extent. Similar to previous reports, *Alphaproteobacteria*, *Gammaproteobacteria*, *Firmicutes*, *Bacteroidetes* and *Actinobacteria* were ubiquitous major groups detected in three coral species. Although *Cyanobacteria* was predominant in the Red Sea corals [19], in *Acropora formosa* and *P. lutea* from Indo-Pacific reefs [18] and in *M. faveolata* from the Caribbean Sea [17], *Cyanobacteria* were rare in the three coral species studied in this work as well as in the results presented by Chen et al [11]. Members of five candidate phyla, including BRC1, OD1, SR1, TM7 and WS3, with the exception of WS3 and TM7 [19], [28], were not previously known to inhabit corals.

In previous studies, the most abundant bacteria of *A. millepora* in the Great Barrier Reef were *Gammaproteobacteria*, *Alphaproteobacteria* and *Betaproteobacteria* or *Deltaproteobacteria* [13], while in this study, *Gammaproteobacteria* and *Firmicutes* and *Deinococcus-Thermus* were dominant in the *A. millepora* affiliated bacterial community. The divergence of habitat between this and previous studies may contribute to the different bacterial communities. Moreover, *Deinococcus-Thermus* has also been observed in stony coral *Pocillopora verrucosa*, *Astreopora myriophthalma* and *S. pistillata* and soft coral *Sarcophyton* sp. from the Red Sea [19]. In contrast to *P. lutea* in Indo-Pacific reefs, *Actinobacteria*, *Bacteroidetes* and *Planctomycetes* were more abundant and *Cyanobacteria* and *Gammaproteobacteria* were less abundant in the *P. lutea*-associated bacterial community in Sanya Bay [18]. Additionally, *Chlorobi* was a major group in the *P. lutea*-associated bacterial community from Sanya Bay, but it was absent in bacterial communities associated with Indo-Pacific *P. lutea* [18]. All of the observations mentioned above suggest that these differences are most likely due to geographical separation and distinct environmental conditions.

### Potential Functional Groups

As coral reefs often reside in nutrient limited waters, nitrogen-fixing microbes are important for compensating the nitrogen deficit in coral holobionts [10]. Several bacteria potentially involved in nitrogen-fixing have been detected in this study, including *Chlorobia*, *Chloroflexi*, *Clostridia* and *Cyanobacteria*. Scleractinian corals are significant contributors to the production of dimethylsufoniopropionate (DMSP) and dimethysulfide (DMS), which are key compounds in the global sulfur cycle [29]. Diverse coral-associated bacteria take part in the degradation of DMSP and DMS. In this study, bacterial groups capable of metabolizing DMSP/DMS, such as *Ruegeria*, *Pseudomonas*, *Acinetobacter*, *Desulfovibrio*, *Flavobacterium*, *Cytophaga*, *Oceanicola* and *Comamonas* [29] were observed in three coral libraries. These diverse and metabolic potential bacterial groups play a crucial role in the biogeochemical cycle. *Actinobacteria* were observed in abundance in coral samples but were rare in seawater. This group may generate a diverse array of antibacterial compounds that protect the coral from pathogens [30]. The coexistence of various potential functional groups should be essential to the coral holobiont. Therefore, the detailed ecological functions of the bacterial groups identified in this study warrant further research.

*Lachnospiraceae* was previously suggested to be a bacterial group for fecal source tracking [31]; however, Newton et al [32] further suggested that the single phylotype Lachno2, which is closely related to the genus *Blautia*, would be a candidate for a host-associated fecal indicator. A high proportion of *Lachnospiraceae* was detected in *G. fascicularis* colony 3, and most of them belonged to an unclassified group. Whether they are related to human fecal bacteria still need further investigated. Additionally, *Escherichia*, which are assumed to be animal-associated bacteria [33], appeared prominently in all *A. millepora* colonies. Because Sanya Bay is a popular tourist spot, the presence of *Lachnospiraceae* and *Escherichia* indicates that we should pay attention to the pollution sources in Sanya Bay, and the real reason for the appearance of these bacteria needs more study.

### Common and Specific Bacterial Communities Associated with Corals

As in sponges and the human gut, the common bacterial community in corals was rather small [34], [35]. The representative sequences of these 22 OTUs shared by three coral species, except 2 OTUs that also observed in seawater, showed ≦98% similarity to sequences in GenBank, most of which were previously found in sponge- or coral-associated microbial communities (Table 2). It appears that these 20 OTUs might be coral-specific bacteria adapted to the coral reef niche. The species-specific community was large in contrast to the common bacterial community. Although bacterial communities associated with corals were grouped into *A. millepora* or *P. lutea* and *G. fascicularis* categories, bacterial composition in each colony varied at the 97% OTU level. This variation has also been detected in the *I*. *palifera* bacterial community [11]. The extensive specificity of coral-associated bacteria might result from varied coral development stages or exterior environments [1], [28]. Previous studies have indicated that the specific bacterial lineages present in individual sponge and human gut microbiomes vary [34], [35]. Turnbaugh et al [35] further proposed that different bacterial species assemblages shared genes among sampled individuals, comprising a “core microbiome” at the genomic level rather than the bacterial lineage level. Different sets of microbial species observed in coral individuals sampled in this study allow for us to speculate that these diverse combinations of species may fulfill the same functional roles required by corals through functional-overlap. Whether this pattern exists in coral-associated bacterial assemblages still needs further global investigation and more direct evidence.

### Conclusions

In this study, bacterial communities associated with corals from the South China Sea were investigated in detail for the first time. The results showed that coral-associated bacteria are highly diverse and are divergent from the seawater bacterial community. Furthermore, the bacterial community associated with *A. millepora* was distinct from *P. lutea* and *G. fascicular*. In comparison with previous studies, bacterial communities associated with *A. millepora* and *P. lutea* in the South China Sea were distinct from those located in the Great Barrier Reef and in Indo-Pacific reefs. It was observed that different coral species share a small common bacterial community, and the composition of the bacterial communities within each colony of each coral species also showed variation. The coexistence of specificity and uniformity reflects the complexity of coral-associated bacterial community and suggests that corals combine the functional bacterial associates in a subtle and sophisticated manner. This study provides novel insights into the complex structure of coral bacterial associates.

## Supporting Information

File S1 **Table S1: Description of the OTUs that contributed to the differences between** ***Porites lutea*** **-** ***Galaxea fascicular*** **and** ***Acropora millepora*** **groups (>1%)**
(DOCX)

File S2 **Table S2: Common bacterial OTUs in** ***Acropora millepora*** **colonies**, Table S3: Common bacterial OTUs in *Porites lutea* colonies, Table S4: Common bacterial OTUs in *Galaxea fascicularis* colonies
(XLSX)
