# Supplementary material for: Highly Heterogeneous Bacterial Communities Associated with the South China Sea Reef Corals Porites lutea, Galaxea fascicularis and Acropora millepora
Source: PLoS One. 2013 Aug 7;8(8):e71301. doi: 10.1371/journal.pone.0071301 (PMC3737133; doi:10.1371/journal.pone.0071301)
Supplement: File S1 — Table S1: Description of the OTUs that contributed to the differences between Porites lutea - Galaxea fascicular and Acropora millepora groups (>1%) (DOCX) [file pone.0071301.s001.docx]

**Table S1.** Description of the OTUs that contributed to the differences between *Porites* *lutea*-*Galaxea fascicular* and *Acropora millepora* groups (> 1%)

| OTU | dominance | classification | **Best BLAST-hit description** | | | | | |
| --- | --- | --- | --- | --- | --- | --- | --- | --- |
|  |  |  | score | coverage % | E-value | identity % | accession # | source |
| OTU1951 | AM | *Deinococcus-Thermus; Deinococci; Thermales; Thermaceae; Meiothermus* | 863 | 99 | 0 | 98 | NR074273 | *Meiothermus silvanus* DSM 9946 from hot spring, Portugal |
| OTU414 | PLGF | *Firmicutes; Clostridia; Clostridiales; Lachnospiraceae* | 839 | 99 | 0 | 96 | FJ654593 | uncultured bacterium from tropical soft coral *Sinularia* sp. subjected to temperature stress |
| OTU6261 | PLGF | *Firmicutes; Clostridia; Clostridiales; Lachnospiraceae* | 922 | 98 | 0 | 99 | FJ654593 | uncultured bacterium from tropical soft coral *Sinularia* sp. subjected to temperature stress |
| OTU3062 | AM | *Deinococcus-Thermus; Deinococci; Deinococcales; Deinococcaceae; Deinococcus* | 880 | 99 | 0 | 99 | HQ876598 | *Deinococcus* sp. Grk4 from Greater Rann of Kutch, India |
| OTU6273 | AM | *Firmicutes; Bacilli; Bacillales; Bacillaceae; Anoxybacillus* | 957 | 100 | 0 | 99 | AY248709 | *Anoxybacillus kestanbolensis* from Kestanbol hot spring, Turkey |

AM represents *A. millepora* group; PLGF represents *P.* *lutea*-*G. fascicular* group.
